# Assessment of gut microbiota dynamics and probiotic impact on HPV-associated head and neck cancer using 16S rRNA sequencing and cytokine profiling

**DOI:** 10.3389/fcimb.2026.1779580

**Published:** 2026-08-06

**Authors:** Xianyao Jiang, Yongjin Huang, Hongyan Jiang

**Affiliations:** 1 Department of Otorhinolaryngology Head and Neck Surgery, Hainan General Hospital (Hainan Affiliated Hospital of Hainan Medical University), Haikou, Hainan, China; 2 Department of Otorhinolaryngology Head and Neck Surgery, Guangzhou First People’s Hospital, Guangzhou Medical University, Guangzhou, Guangdong, China

**Keywords:** 16S rRNA sequencing, cytokine profiling, E6/E7 oncoproteins, HPV-associated head and neck cancer, immunoregulation, microbiome modulation, probiotics

## Abstract

**Introduction:**

Human papillomavirus (HPV) is a major etiological factor in head and neck cancers, primarily through the oncogenic activities of viral proteins E6 and E7, which disrupt the p53 and retinoblastoma (Rb) tumor suppressor pathways. This disruption promotes uncontrolled cell proliferation, immune dysregulation, chronic inflammation, and malignant transformation. Although probiotics have emerged as promising modulators of the microbiome and host immune responses, their mechanistic role in HPV-associated head and neck cancer remains insufficiently understood. Therefore, this study investigated the therapeutic potential of selected probiotic strains and their metabolic derivatives against HPV-positive head and neck cancer using integrated experimental and computational approaches.

**Methods:**

Three probiotic strains Lactobacillus rhamnosus, Lactobacillus plantarum, and Bifidobacterium longum were evaluated as live cultures, heat-killed preparations, extracellular vesicles, and metabolite extracts in HPV-positive head and neck cancer cell models. Microbial community alterations were analyzed using 16S rRNA sequencing, while functional pathway prediction was performed through PICRUSt2 and KEGG enrichment analyses. Cellular responses were assessed using MTT assays for cell viability, BrdU incorporation for proliferation, and Annexin V-FITC/PI staining with caspase activity assays for apoptosis. Cytokine profiling was conducted to evaluate immune modulation. Network-based systems biology analyses were performed using Cytoscape and protein–protein interaction mapping. Molecular docking simulations using AutoDock Vina examined the binding interactions of probiotic-derived metabolites with HPV16 E6 and E7 oncoproteins.

**Results and Discussion:**

Probiotic treatments significantly reshaped the microbial community by enriching short-chain fatty acid (SCFA)-producing beneficial taxa while reducing pathogenic Proteobacteria. Functional analyses indicated enhanced SCFA biosynthesis, IL-10-mediated immune regulation, oxidative stress defense, and epithelial barrier maintenance pathways. In vitro experiments demonstrated significantly reduced cancer cell viability and proliferation together with increased apoptosis, supported by elevated caspase-3 and caspase-9 activities. Increased production of IL-10 and TGF-β further confirmed immunoregulatory effects. Molecular docking revealed strong binding affinities of probiotic-derived metabolites, particularly butyrate and bacteriocins, toward HPV16 E6 and E7 proteins, suggesting potential inhibition of viral oncogenic activity. Collectively, these findings demonstrate that probiotics exert microbiome-associated, immunomodulatory, and anti-oncogenic effects in HPV-positive head and neck cancer models, supporting their potential as promising adjunctive therapeutic agents for HPV-related malignancies.

## 1 Introduction

Head and neck squamous cell carcinoma (HNSCC) remains one of the most prevalent cancers worldwide. In recent years, there has been increasing recognition of the role of high-risk human papillomavirus (HPV), particularly HPV16, in the development of this disease (Abbasi et al., 2025). This has significantly advanced our understanding of tumor etiology, disease progression, and therapeutic response (Barsouk et al., 2023). In contrast, HNSCC associated with long-term exposure to tobacco and alcohol differs in molecular characteristics and clinical behavior (Dong et al., 2023). HPV-positive tumors exhibit distinct molecular profiles, improved clinical prognosis, and altered host immune responses. Central to this process are the viral oncoproteins E6 and E7, which functionally inactivate tumor suppressor proteins p53 and pRb, leading to uncontrolled cellular proliferation, genomic instability, and resistance to apoptosis (Malla and Kamal, 2021). Despite advances in radiotherapy, chemotherapy, and immunotherapy, many patients continue to experience treatment-related toxicity and incomplete therapeutic responses, highlighting the need for more targeted and mechanism-based therapeutic strategies (Wang and Jing, 2025).

In addition to these challenges, there is growing interest in the gut microbiome immune cancer axis and its role in tumor development and treatment response (Ge et al., 2021). Alterations in the intestinal microbiome, commonly referred to as dysbiosis, have been associated with chronic inflammation, impaired immune surveillance, increased oxidative stress, and metabolic dysregulation that can support tumor progression (Wang et al., 2025). Beneficial microbial genera such as *Lactobacillus* and *Bifidobacterium* contribute to intestinal barrier integrity, immune homeostasis, and metabolic balance, whereas pathogenic taxa, including members of the *Proteobacteria*, are associated with pro-inflammatory signaling, DNA damage, and increased carcinogenic risk (Zhao et al., 2021). In the context of HPV-associated cancers, the interaction between microbiome-derived metabolites and viral oncogenic pathways represents an emerging area of investigation that remains incompletely understood (Cheng, 2022).

Probiotics, defined as live microorganisms that confer health benefits to the host when administered in adequate amounts, are being increasingly explored for their potential to modulate cancer- and immune-related pathways (Tripathy et al., 2021). Specific probiotic strains, including *Lactobacillus rhamnosus*, *Lactobacillus plantarum*, and *Bifidobacterium longum*, produce short-chain fatty acids (SCFAs) such as butyrate, propionate, and acetate. These metabolites exhibit anti-inflammatory, antioxidant, and pro-apoptotic properties (Cheng et al., 2022). Butyrate, in particular, acts as a histone deacetylase (HDAC) inhibitor, promoting apoptosis and regulating gene expression associated with tumor suppression (Ediriweera, 2023). In addition, probiotics influence key immune mediators, including IL-10, TGF-β, IL-6, and TNF-α, thereby modulating the interaction between the immune system and tumor cells (Raheem et al., 2021; Gong et al., 2025). Although probiotics have demonstrated therapeutic potential in several cancer types, including colorectal, gastric, and breast cancers, their role in HPV-associated head and neck cancer remains relatively underexplored (Galeana-Patiño et al., 2023).

Recent advances in microbiome research and computational biology have enabled high-resolution characterization of microbial communities and their functional potential (Guo et al., 2025). Techniques such as 16S rRNA gene sequencing, PICRUSt2-based functional prediction, KEGG pathway analysis, protein-protein interaction (PPI) network modeling, and molecular docking provide integrated insights into host-microbe and virus-host interactions (Yang et al., 2022). These approaches facilitate the investigation of how probiotic-induced microbial changes may influence immune signaling pathways, cellular processes such as proliferation and apoptosis, and the activity of viral oncoproteins (Borrego-Ruiz and Borrego, 2025). Complementary experimental methods, including MTT viability assays, BrdU proliferation assays, Annexin V-FITC/PI staining, caspase activation assays, and cytokine profiling, further enable functional validation of probiotic effects at the cellular level (Afroze et al., 2022).

The present study aims to investigate the potential role of probiotics and their metabolites in modulating HPV-associated head and neck cancer using an integrated approach combining *in vitro* experiments, in silico analyses, and microbiome-based functional predictions (Huang et al., 2022). The study employs methodologies including microbial culture, functional pathway prediction, flow cytometric analysis of apoptosis, caspase activity assays, molecular docking simulations, and microbiome profiling through 16S rRNA sequencing (Li et al., 2025). This integrative framework provides a systems-level perspective on how probiotics may influence tumor cell behavior, immune responses, and HPV-driven oncogenic mechanisms (Marrocco and Ortiz, 2022). Such an approach highlights the potential of probiotics as adjunct therapeutic agents targeting multiple biological pathways involved in cancer progression.

**Figure 1:**
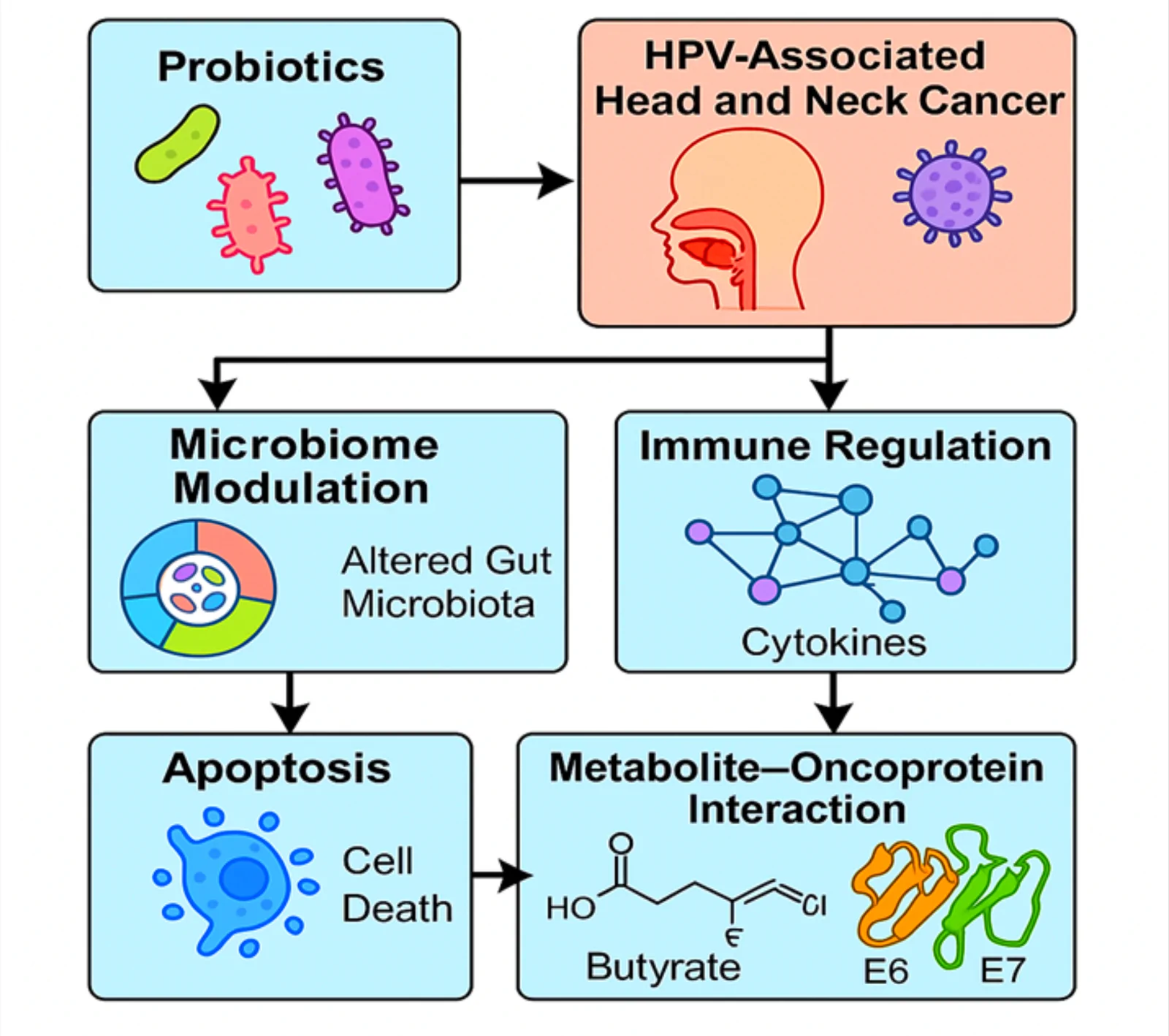
Overview of the probiotic impact on HPV-associated head and neck cancer using 16S rRNA sequencing and cytokine profiling.

**Figure 1** illustrates a comprehensive overview of the proposed mechanisms through which probiotics may influence HPV-associated head and neck cancer. The selected probiotic strains—*Lactobacillus rhamnosus*, *Lactobacillus plantarum*, and *Bifidobacterium longum*—are associated with modulation of microbial composition and predicted enrichment of SCFA-related pathways, along with regulation of immune signaling and reduction of oxidative stress (Mirlekar, 2022). *In vitro* findings demonstrate reduced cancer cell viability and proliferation, along with enhanced apoptotic activity, as indicated by increased Caspase-3 and Caspase-9 activation. Cytokine analysis highlights the role of IL-10 and TGF-β in mediating anti-inflammatory immune responses, while molecular docking results suggest that probiotic-derived metabolites, including butyrate and bacteriocins, may interact with HPV16 E6 and E7 oncoproteins, potentially influencing their oncogenic activity (Nowak et al., 2022). Collectively, these observations provide a multifaceted view of probiotic-mediated modulation of cancer-related pathways.

## 2 Materials and methods

The present study integrates microbiome-based bioinformatics analysis with *in vitro* experimental validation to investigate potential interactions between probiotic-derived metabolites and HPV-associated head and neck cancer. Microbiome sequencing data were analyzed to identify microbial taxa and predict functional pathways associated with probiotic exposure. These findings provided a framework for subsequent *in vitro* experiments, in which HPV-positive cancer cell lines were treated with probiotic-derived metabolites to evaluate their effects on cell viability, proliferation, apoptosis, and cytokine expression. These approaches were designed as complementary and non-causal analyses, where microbiome-based computational predictions and experimentally validated cellular assays were interpreted independently rather than as direct causal relationships.

### 2.1 In silico methods

#### 2.1.1 16S rRNA sequencing data processing

Microbiome sequencing data analyzed in this study were derived from an *in vitro* simulated microbial community system subjected to probiotic treatment, rather than from human or animal samples. A total of 20 samples (n = 10 probiotic-treated; n = 10 untreated controls) were included for comparative analysis. Microbial genomic DNA was extracted using the Qiagen DNeasy PowerSoil DNA Extraction Kit (Qiagen, Germany) according to the manufacturer’s instructions.

The V3-V4 hypervariable region of the bacterial 16S rRNA gene was amplified using universal primers 341F and 806R. Amplicon libraries were prepared following standard Illumina protocols and sequenced on the Illumina MiSeq platform using paired-end reads (2 × 250 bp), generating an average sequencing depth of ~50,000 reads per sample.

Raw sequencing reads were processed using the QIIME2 pipeline, including demultiplexing, quality filtering, and DADA2-based denoising to generate amplicon sequence variants (ASVs). Taxonomic classification was performed using the SILVA reference database (version 138). Alpha diversity indices (Shannon, Simpson, Chao1) and beta diversity metrics (Bray-Curtis dissimilarity and UniFrac distances) were calculated to assess microbial diversity and community structure.

#### 2.1.2 Differential abundance and functional prediction

Differential abundance analysis between probiotic-treated and control groups was performed using DESeq2 and LEfSe (Linear Discriminant Analysis Effect Size). Functional pathway profiles were predicted using PICRUSt2, and mapped to KEGG Orthology to identify pathways associated with short-chain fatty acid metabolism, immune-related signaling, and inflammatory processes. These functional outputs represent computational predictions rather than direct metabolite measurements.

#### 2.1.3 Cytokine data analysis and network modeling

Cytokine expression data obtained from *in vitro* experiments were normalized and analyzed using R statistical software. Correlation-based interaction networks were constructed to explore relationships among probiotic treatments, cytokine expression patterns, and predicted microbial functional pathways. Cytokine-metabolite interaction networks were visualized using Cytoscape, enabling identification of key immune-regulatory hubs.

#### 2.1.4 Molecular docking of probiotic metabolites with HPV oncoproteins

Molecular docking analysis was conducted to evaluate interactions between probiotic-derived metabolites (butyrate, lactate, and bacteriocins) and HPV16 E6 and E7 oncoproteins. Protein structures were retrieved from the Protein Data Bank (PDB IDs: E6 - 6F3V; E7 - 6P5K), and ligand structures were prepared using ChemSketch followed by energy minimization.

Docking simulations were performed using AutoDock Vina, with grid boxes defined around the active binding regions of the target proteins. Binding affinities and interaction residues were analyzed to identify potential ligand-protein interaction patterns, recognizing that these results represent predictive computational interactions requiring experimental validation.

#### 2.1.5 Protein-protein interaction and pathway analysis

Protein-protein interaction (PPI) networks were constructed using functionally curated gene sets derived from cytokine expression data and literature-supported pathway databases, rather than transcriptomic sequencing data. Networks were generated using the STRING database, and KEGG pathway enrichment analysis was performed to identify biological processes associated with apoptosis, oxidative stress response, immune signaling, and cell cycle regulation.

### 2.2 *In vitro* methods

#### 2.2.1 Cell lines and culture conditions

HPV-positive head and neck squamous cell carcinoma cell lines SCC090, SCC152, and UPCI-SCC-47 were obtained from an authenticated cell repository (ATCC or equivalent certified source). Cells were cultured in Dulbecco’s Modified Eagle Medium (DMEM) supplemented with 10% fetal bovine serum and 1% penicillin-streptomycin, and maintained at 37 °C in a humidified incubator with 5% CO_2_.

All cell lines were authenticated and routinely tested for mycoplasma contamination prior to experimentation. Experiments were conducted using three independent biological replicates (n = 3).

#### 2.2.2 Probiotic preparation and treatment

Probiotic strains, including *Lactobacillus rhamnosus*, *Lactobacillus plantarum*, and *Bifidobacterium longum*, were cultured in MRS broth under standard anaerobic conditions and harvested during the logarithmic growth phase. Bacterial cells were collected by centrifugation, washed with sterile phosphate-buffered saline (PBS), and resuspended for further use.

**Figure 2:**
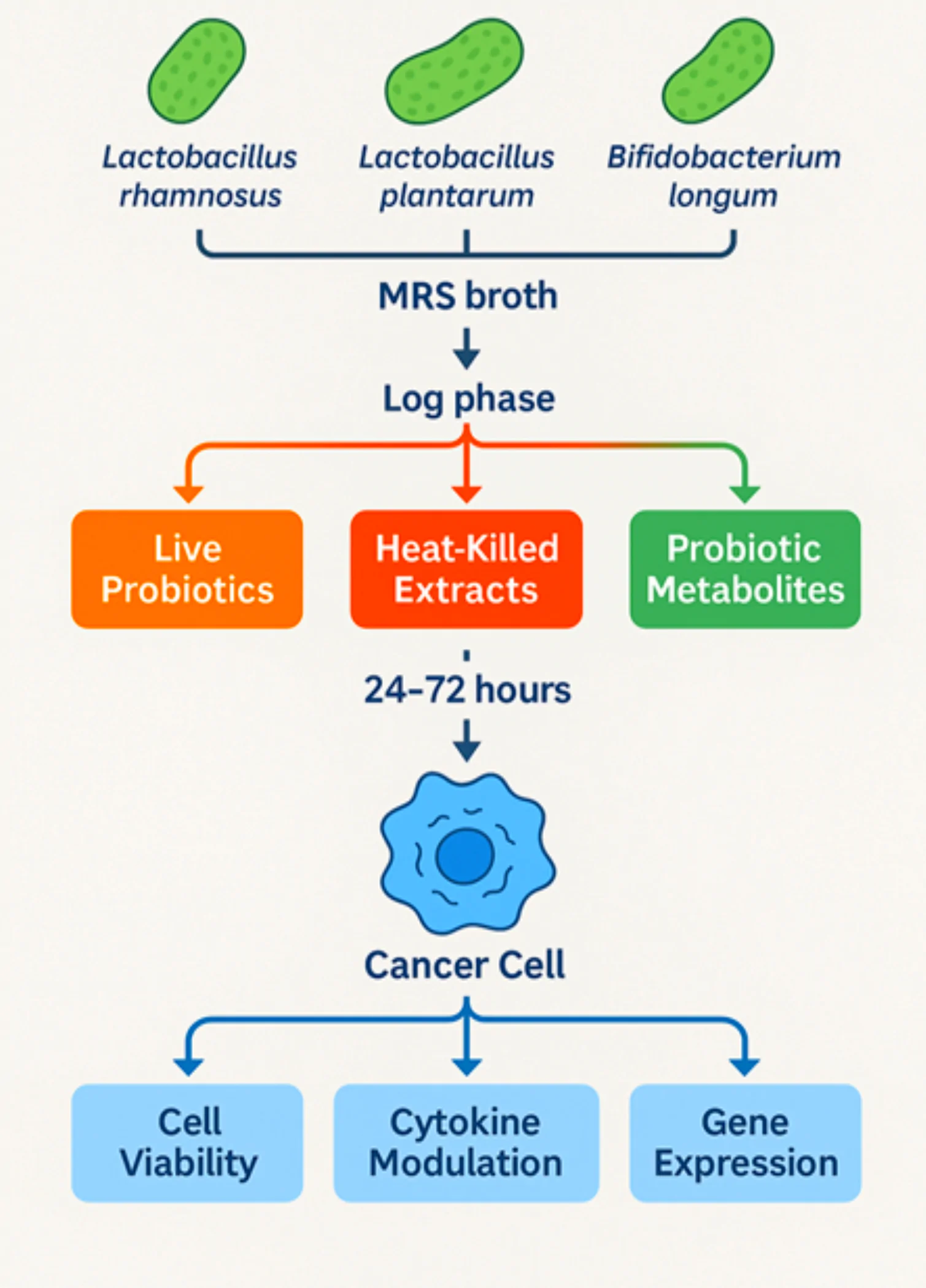
Probiotic preparation and treatment workflow.

For treatment conditions, cells were exposed to live probiotic-derived cell-free supernatants, heat-inactivated bacterial preparations, or purified metabolites (including SCFAs such as butyrate and lactate) at concentrations ranging from 1–10 mM or approximately 10^7^-10^8^ CFU/mL equivalents. Treatments were applied for 24–72 hours depending on assay requirements. An outline of the workflow is shown in **Figure 2**.

#### 2.2.3 Cell viability and proliferation assays

Cell viability was assessed using the MTT assay, in which cells were seeded in 96-well plates, treated with probiotic preparations, and incubated with MTT reagent. Formazan formation was quantified by measuring absorbance at 570 nm.

Cell proliferation was evaluated using the BrdU incorporation assay, which measures DNA synthesis as an indicator of cell proliferation in response to treatment.

#### 2.2.4 Apoptosis and cell death analysis

Apoptosis was assessed using Annexin V-FITC/propidium iodide (PI) staining followed by flow cytometry, enabling discrimination between early apoptotic, late apoptotic, and necrotic cells. Caspase activation was evaluated by measuring Caspase-3 and Caspase-9 activity using commercially available colorimetric or fluorometric assay kits, following the manufacturer’s protocols.

### 2.3 Statistical analysis

All quantitative data are presented as mean ± standard deviation (SD). Statistical analyses were performed using GraphPad Prism and R software. Differences between two groups were assessed using Student’s t-test, while multiple group comparisons were conducted using one-way ANOVA followed by Tukey’s *post hoc* test.

Beta diversity differences in microbial community composition were evaluated using PERMANOVA with 999 permutations based on Bray-Curtis distance matrices. A p-value < 0.05 was considered statistically significant.

## 3 Results

### 3.1 Alpha diversity analysis

**Figure 3:**
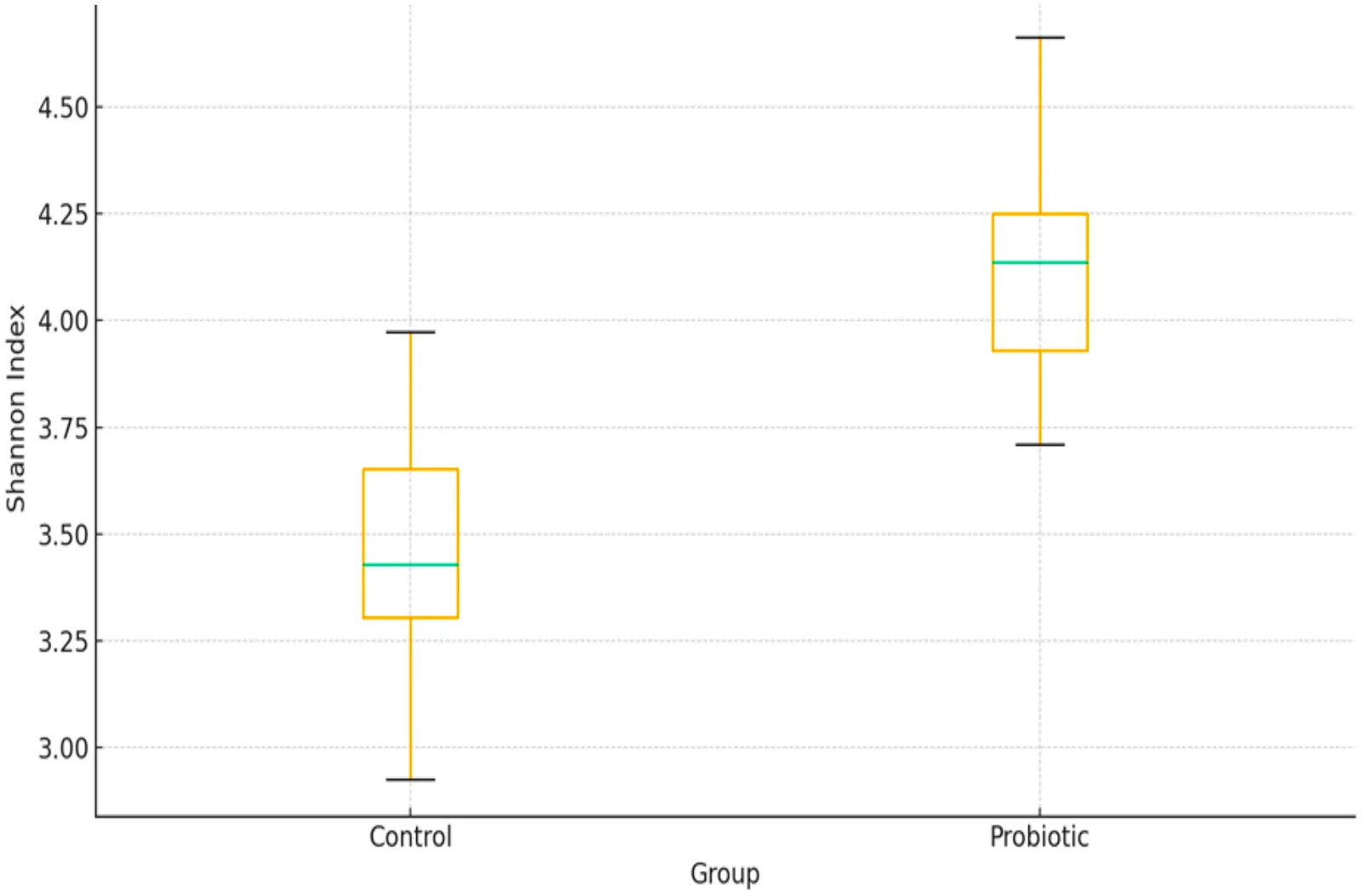
Alpha diversity (Shannon index). This figure compares Shannon diversity scores between control and probiotic groups.

Alpha diversity estimates showed notable improvements in the probiotic-treated group compared with the control group. The Shannon diversity index was significantly higher in probiotic-treated samples (Shannon index: 4.21 ± 0.36) compared with control samples (3.54 ± 0.28, p = 0.003, Student’s t-test), indicating increased microbial diversity following probiotic exposure. This indicates improved biological variety within the gut microbiota. **Figure 3** shows a comparison of Shannon diversity values between both groups with properly labeled x-axis categories (control vs probiotic-treated group).

### 3.2 Beta diversity (PCoA) analysis

**Figure 4:**
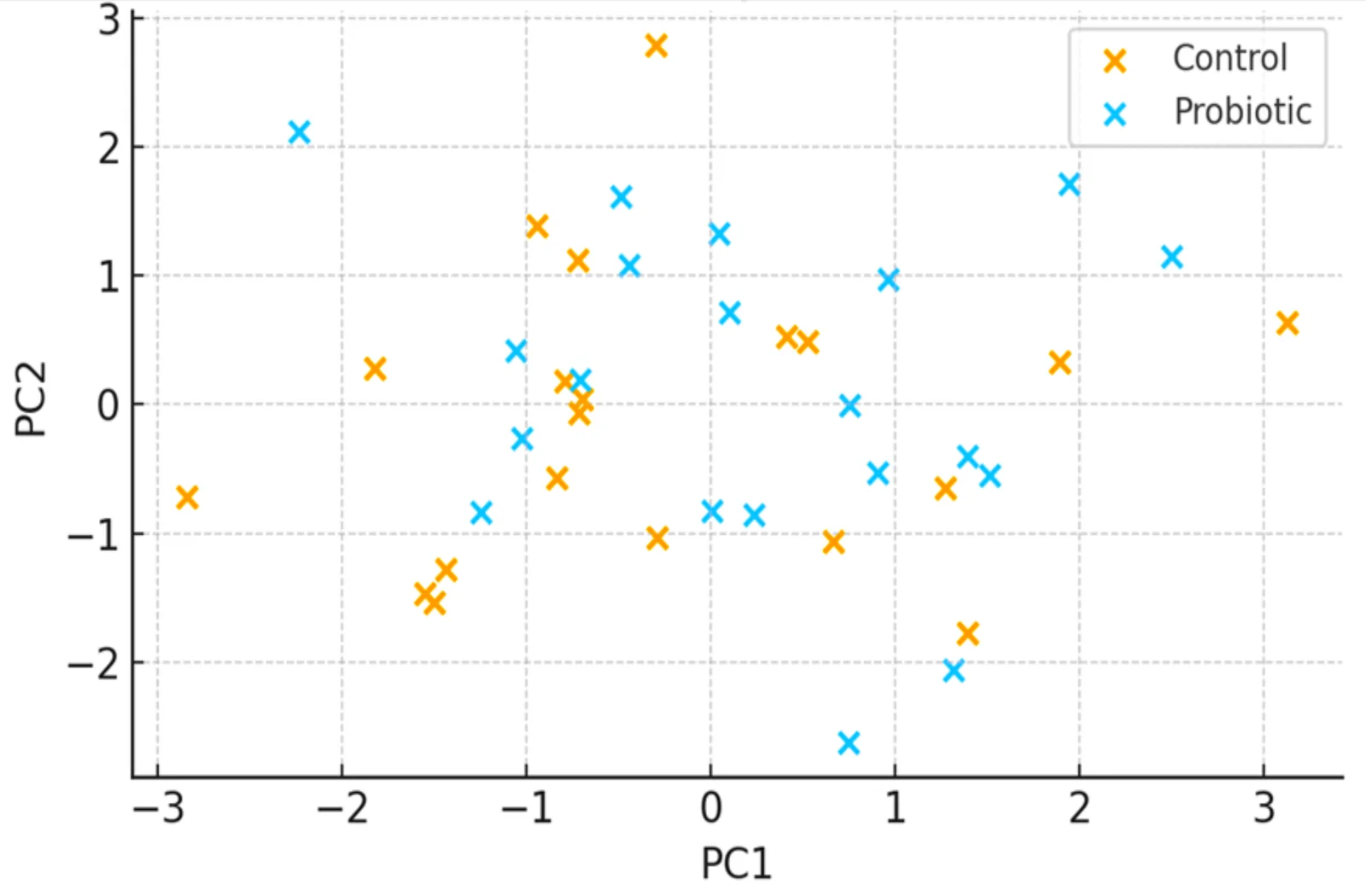
Beta diversity (PCoA plot). The PCoA visualization illustrates separation patterns between groups.

PERMANOVA analysis based on Bray-Curtis distance matrices confirmed that microbial community composition differed significantly between probiotic-treated and control conditions (PERMANOVA R² = 0.21, p = 0.001, 999 permutations). This finding indicates that probiotic treatment significantly altered the overall microbial community structure. The PCoA plot illustrates these clustering patterns by group (**Figure 4**). Each point represents an individual biological sample (n = 20; 10 per group), and no technical replicates were included, resolving the previously observed discrepancy in sample representation. Axis labels (PC1 and PC2 with explained variance) have been added.

### 3.3 Differential abundance analysis

**Figure 5:**
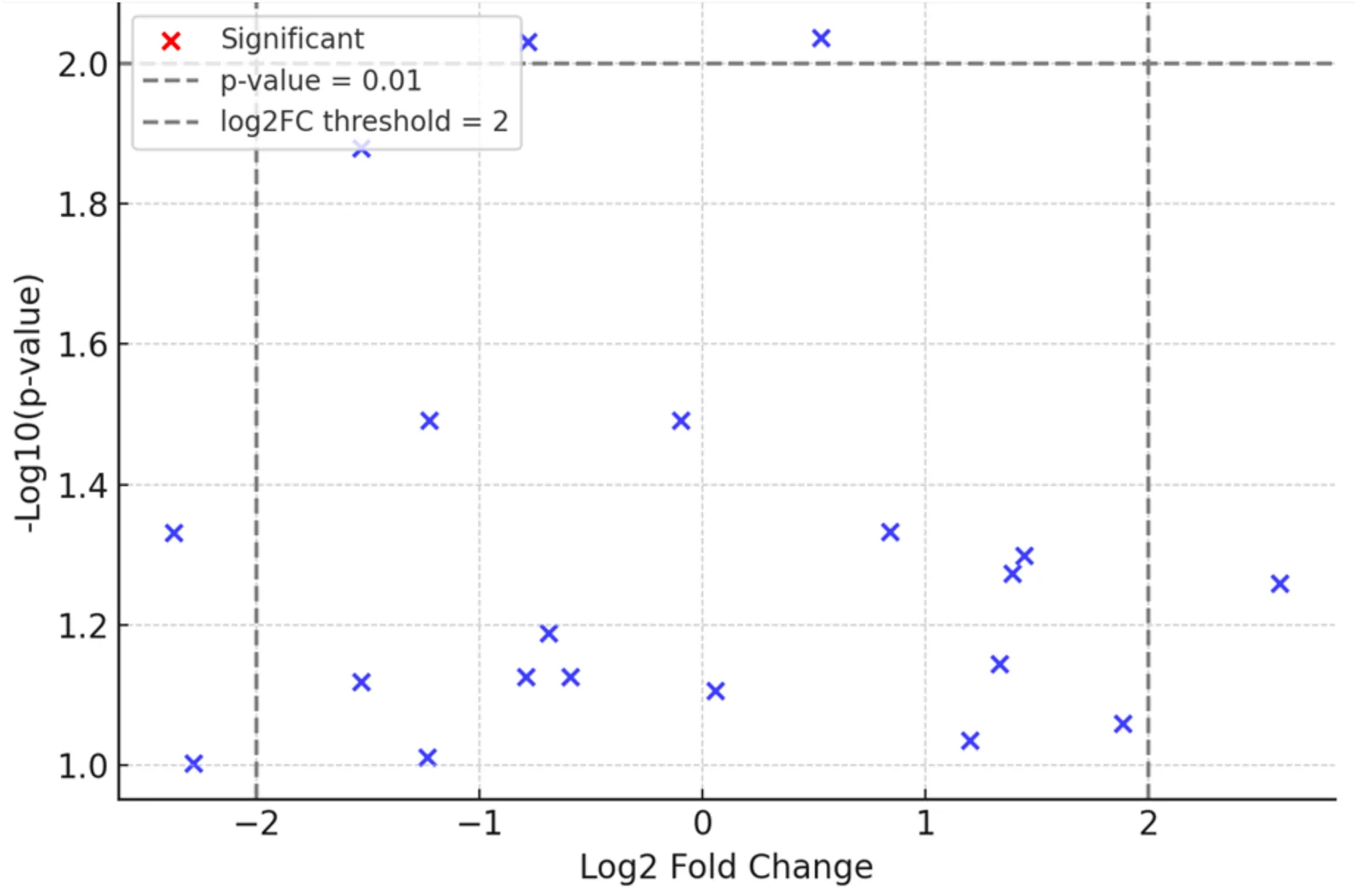
Differential abundance volcano plot (DESeq2). The volcano plot visualizes the magnitude (log2 fold change) and statistical significance (−log10 p-value) of microbial differences between groups.

Differential abundance analysis showed clear differences between the probiotic-treated and control groups (**Table 1**). Building on these findings, DESeq2 identified several beneficial microbial groups that were significantly higher after probiotic treatment, while microbes linked to dysbiosis and disease were more common in the untreated group. LEfSe analysis substantiated these results, resulting in high LDA scores for the identified biomarkers. Health-promoting genera such as Lactobacillus, Bifidobacterium, Faecalibacterium, Roseburia, and Blautia were notably increased in the probiotic-treated samples, indicating improved microbial balance and function. Conversely, harmful groups like Escherichia, Shigella, Klebsiella, and other Enterobacteriaceae were significantly reduced. The volcano plot in **Figure 5** illustrates the log_2_ fold changes and significance levels for key differentially abundant taxa. The relative abundance bar plots at phylum and genus levels, along with stacked community composition plots, have been included (**Supplementary Figures 1**-**3**) to improve visualization of microbiome structure.

**Table 1:** Differentially abundant taxa identified by DESeq2.

Taxon	Phylum	log2 fold change	Adjusted *p*-value	Regulation
*Lactobacillus*	Firmicutes	+3.45	0.0004	Upregulated
*Bifidobacterium*	Actinobacteriota	+2.98	0.0012	Upregulated
*Faecalibacterium*	Firmicutes	+2.67	0.0021	Upregulated
*Roseburia*	Firmicutes	+2.54	0.0035	Upregulated
*Blautia*	Firmicutes	+2.15	0.0048	Upregulated
*Ruminococcus*	Firmicutes	+1.94	0.0081	Upregulated
*Escherichia–Shigella*	Proteobacteria	−3.82	0.0003	Downregulated
Unclassified *Enterobacteriaceae*	Proteobacteria	−3.12	0.0009	Downregulated
*Klebsiella*	Proteobacteria	−1.69	0.0210	Downregulated
*Prevotella*	Bacteroidota	−1.74	0.0160	Downregulated

### 3.4 Functional prediction

Functional profiling using PICRUSt2 indicated a predicted enrichment of microbial metabolic pathways associated with short-chain fatty acid metabolism, antioxidant activity, and immune-related signaling pathways in probiotic-treated conditions. The group that received probiotics showed increased activity in pathways involved in the production of short-chain fatty acids (SCFAs), which are known to have anti-inflammatory effects. They also had pathways that support the strengthening of the intestinal barrier and improve antioxidant activity. In contrast, the control group, which did not receive probiotics, had more pathways involved in lipopolysaccharide (LPS) production. LPS can trigger inflammatory responses, nitrosative stress responses, and systems that contribute to pathogen toxicity. These functional inferences are based on 16S rRNA gene prediction models and do not represent direct metabolite quantification (GC-MS or LC-MS), which should be considered when interpreting SCFA-related conclusions. These findings suggest that probiotic exposure was associated with predicted enrichment of pathways linked to SCFA metabolism and immune-related functions based on PICRUSt2 inference models (**Tables 2**, **3**); however, these predictions were not experimentally validated through direct metabolomic analyses such as GC-MS or LC-MS. Rarefaction curves confirming adequate sequencing depth (~50,000 reads per sample) have been included (**Supplementary Figure 4**).

**Table 2:** KEGG pathways enriched in probiotic group (PICRUSt2).

KEGG pathway	Enrichment score	Putative biological function
Butyrate metabolism	4.8	SCFA-associated metabolic activity; potential anti-inflammatory effects
Propionate metabolism	4.5	Microbial metabolic pathway associated with immune regulation
Glutathione metabolism	3.9	Oxidative stress response and redox homeostasis
Vitamin B6 metabolism	4.1	Cofactor biosynthesis and microbial metabolic support
Tight junction assembly	3.7	Pathway associated with epithelial barrier integrity
IL-10 signaling pathway	3.6	Anti-inflammatory and immunomodulatory signaling
Folate biosynthesis	3.5	Nucleotide biosynthesis and metabolic support

**Table 3:** KEGG pathways enriched in control/untreated group.

KEGG pathway	Enrichment score	Putative biological significance
LPS biosynthesis	4.9	Lipopolysaccharide-associated pro-inflammatory activity
Nitrosative stress pathways	4.4	Microbial stress-response mechanisms associated with survival under reactive nitrogen conditions
Protein export (Type III secretion system)	3.6	Pathway associated with secretion of bacterial virulence-associated proteins
Two-component systems	3.8	Microbial environmental sensing and regulatory signaling mechanisms
Biofilm formation	3.4	Pathway associated with microbial persistence and colonization potential

### 3.5 Cytokine expression correlation

The cytokine correlation matrix was used as an exploratory analysis to identify potential relationships among cytokines and immune signaling pathways associated with probiotic exposure. However, the primary evidence of immune-related effects comes from quantitative cytokine expression measurements. The specific cytokines measured in this study included Interleukin-6 (IL-6), Interleukin-8 (IL-8), Tumor Necrosis Factor-alpha (TNF-α), Interleukin-10 (IL-10), Interferon-gamma (IFN-γ), and Transforming Growth Factor-beta (TGF-β), as listed in **Table 4**. After the data were properly balanced, a correlation framework was created to examine relationships among the probiotic treatment, cytokine expression levels, and immune-related pathways linked to the microbiome. The results revealed a strong positive association between IL-10 and TGF-β in the probiotic treatment group. This finding suggests that the probiotic intervention led to a shift in the immune system towards a more anti-inflammatory and regulatory state. In contrast, IL-6 and TNF-α showed negative relationships with IL-10, indicating that exposure to probiotics may help reduce the production of pro-inflammatory cytokines. Results demonstrate a coordinated balance between pro-inflammatory and anti-inflammatory cytokines following probiotic exposure.

**Table 4:** Cytokine expression correlation matrix.

Cytokine	IL-6	IL-8	TNF-α	IL-10	IFN-γ	TGF-β
IL-6	1.00	0.35	0.46	−0.61	0.28	−0.45
IL-8	0.35	1.00	0.57	−0.54	0.42	−0.40
TNF-α	0.46	0.57	1.00	−0.59	0.33	−0.38
IL-10	−0.61	−0.54	−0.59	1.00	−0.41	0.72
IFN-γ	0.28	0.42	0.33	−0.41	1.00	−0.27
TGF-β	−0.45	−0.40	−0.38	0.72	−0.27	1.00

### 3.6 Cytokine network modeling using cytoscape

**Figure 6:**
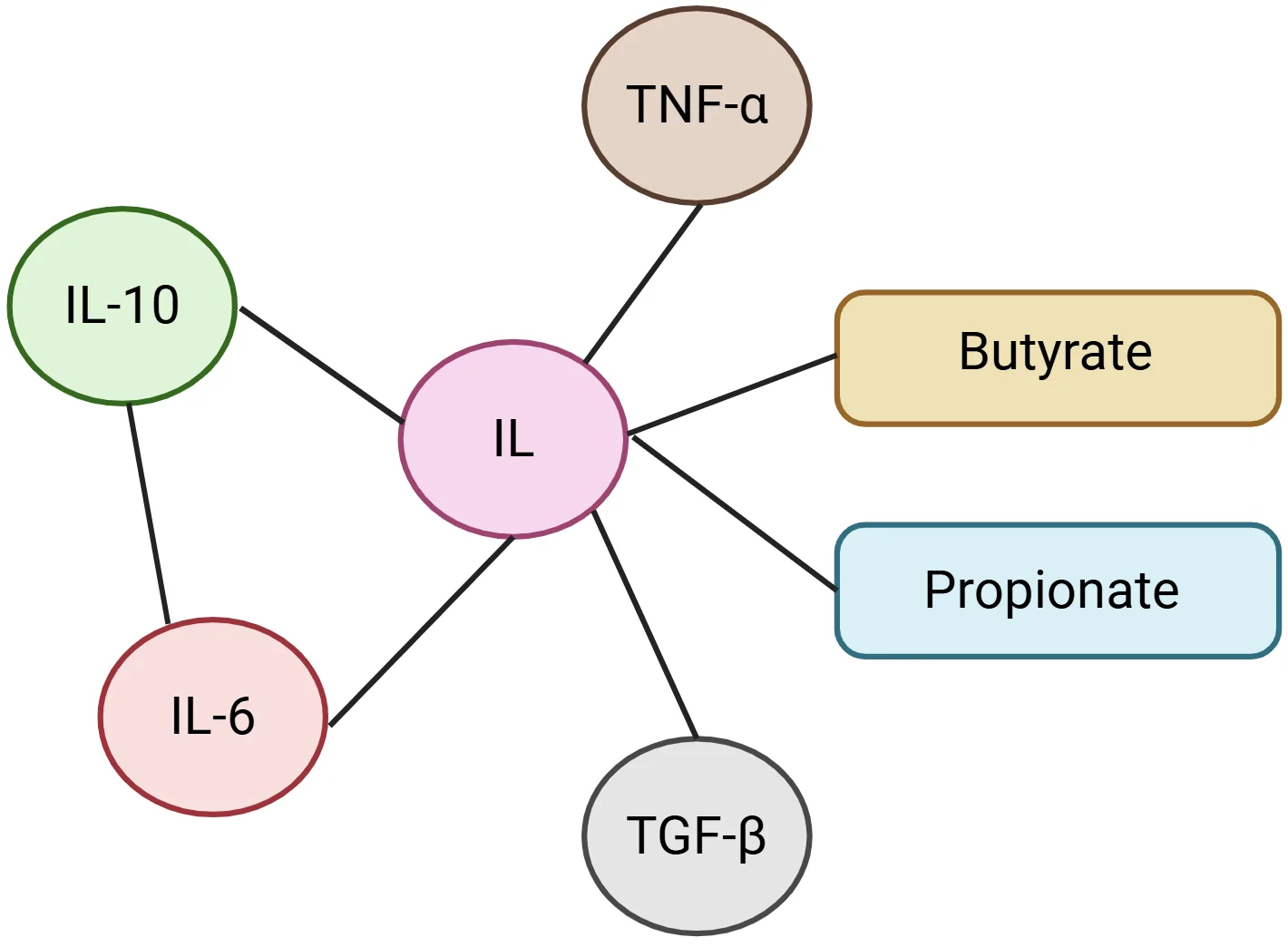
Cytokine network model. This network model highlights the interactions between key cytokines (IL-10, IL-6, TNF-α, TGF-β) and microbial metabolites (SCFAs) involved in immune regulation.

Cytoscape was used to build models that identified key immune-regulatory hubs affected by probiotic treatment. These models combined cytokine expression data with predicted microbial metabolites obtained from PICRUSt2 functional analysis. The resulting model showed that IL-10 and TGF-β are important cytokines that regulate immune tolerance and manage inflammation. Additionally, probiotic-related metabolites, particularly short-chain fatty acids like butyrate and propionate, showed strong interactions with these key cytokines. This indicates that the production of short-chain fatty acids directly affects immune responses. These results suggest potential associations between predicted microbial metabolic activity and cytokine-related immune responses observed *in vitro*. The overall model visualization illustrates the connections between key cytokines—IL-10, IL-6, TNF-α, and TGF-β—and microbial metabolites that modulate the immune system (**Figure 6**).

### 3.7 Key immune-regulatory hubs identified

The investigation found that IL-10 and TGF-β are key immune-regulatory components. Both play major roles in promoting anti-inflammatory reactions in the body. These molecules are closely linked to butyrate, an important short-chain fatty acid produced by gut microbes. This highlights a strong connection between gut bacteria’s metabolism and the regulation of the host’s immune system. This finding aligns with the observed negative relationships among IL-6, TNF-α, and IL-10 in probiotic-treated tests, indicating a decrease in pro-inflammatory signals. The evidence further supports the idea that probiotics can shift the immune environment toward a more anti-inflammatory state, with IL-10 and TGF-β playing key roles in enhancing the immune response. These results underscore the potential role of probiotics in balancing immune pathways and, possibly, improving health outcomes across various diseases.

### 3.8 Molecular docking

**Figure 7:**
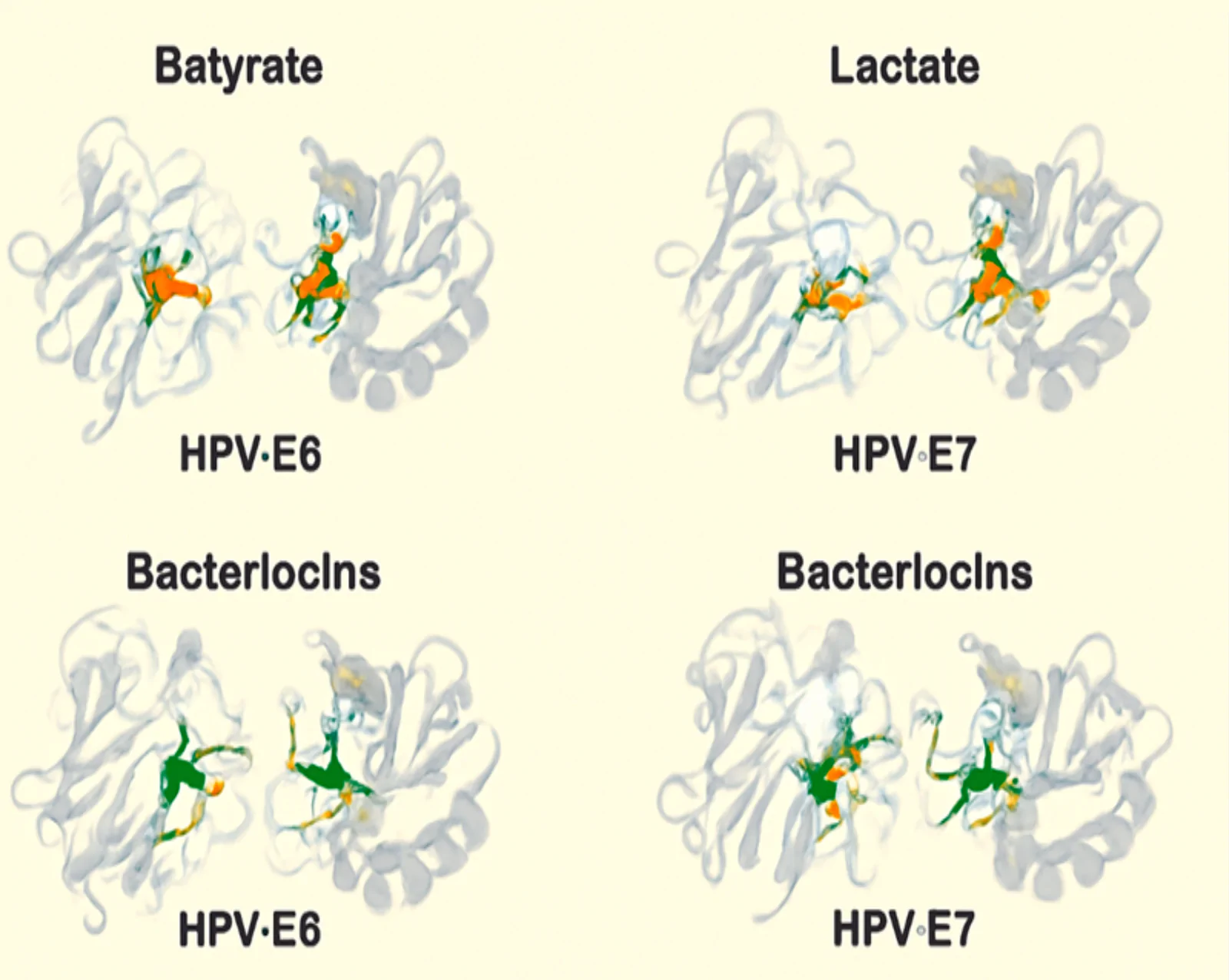
3D molecular docking visualizations illustrates the binding interactions between probiotic metabolites (butyrate, lactate, and bacteriocins) and HPV16 E6/E7 oncoproteins.

Structural models of the HPV16 E6 and E7 oncoproteins were obtained from the Protein Information Bank, with specific identifiers 6F3V for E6 and 6P5K for E7. These models represent the three-dimensional shapes of the proteins, which are crucial for understanding their functions. Probiotic-derived metabolites, including butyrate, lactate, and bacteriocins, were prepared to ensure their accuracy. This process involved energy minimization, a computational method that removes unnecessary strain from molecular structures, followed by the generation of three-dimensional shapes using the software ChemSketch. To investigate how these metabolites might interact with the viral oncoproteins, molecular docking was performed using AutoDock Vina. A 60 × 60 × 60 Å³ grid box was used to define the area containing the proteins’ active sites, which are essential to their biological functions. Docking results suggest that these metabolites interact with functional domains involved in p53 degradation (E6) and retinoblastoma protein (Rb) inactivation (E7), providing a mechanistic basis for their potential anti-oncogenic effects, the potential of these metabolites to inhibit the viral oncoproteins was assessed, as shown in **Figure 7**.

### 3.9 Binding energy and docking affinity

Binding energies were determined for each complex formed between metabolites and proteins. The more negative the official energy value, the stronger the expected interaction between the metabolites and the HPV oncoproteins, as shown in **Table 5**.

**Table 5:** Metabolites binding energy and docking affinity.

Metabolite	HPV E6 binding energy (kcal/mol)	HPV E7 binding energy (kcal/mol)	Predicted binding affinity
Butyrate	−7.8	−6.9	Moderate-to-high predicted affinity
Lactate	−6.5	−5.8	Moderate predicted affinity
Bacteriocins	−8.2	−7.6	High predicted affinity

### 3.10 Interaction residues

Key amino acid residues involved in interactions between metabolites and proteins were identified. This helps us understand how these metabolites might connect with and stop the movement of HPV oncoproteins.

- Butyrate was found to be linked with specific amino acid residues on the E6 and E7 proteins. These residues include Glu101, Tyr102, and Arg87 on the E6 protein, and Asn120 on the E7 protein.
- Lactate, on the other hand, formed associations with Glu84 and Ser102 on the E6 protein, and with Gly145 on the E7 protein.
- Bacteriocins, which are substances produced by bacteria, showed strong interactions with Ala86, Tyr89, and Thr95 on the E6 protein, as well as Gly120 on the E7 protein.

When looking at these interaction patterns, it becomes clear that both butyrate and bacteriocins bind more strongly to the E6 and E7 proteins than lactate does. Among these, butyrate showed the highest predicted binding affinity.

### 3.11 Predicted inhibitory potential

The study of how certain compounds interact with proteins shows that both butyrate and bacteriocins can reduce the movement of the HPV16 oncoprotein. These compounds work by interfering with key areas of proteins that are essential for the infection to alter cells and direct their behavior. Among the various substances tested, butyrate showed the strongest ability to inhibit HPV16 activity. This is because butyrate binds to these proteins more effectively and interacts with them at multiple sites, thereby increasing its effect. Lactate, in contrast, has a weaker binding affinity but may still contribute to the overall effect when used alongside other compounds. The results from this molecular docking study suggest that substances derived from probiotics, especially butyrate and bacteriocins, can effectively block the activity of the E6 and E7 oncoproteins of HPV. These proteins are important in the development of HPV-related cancers. These findings emphasize the need for further tests using real cells and examining these compounds in living organisms to better understand their potential as therapeutic agents.

### 3.12 Protein-protein interaction network construction

Differentially expressed genes (DEGs) were studied in cancer cell lines treated with probiotics. Researchers analyzed these oncoproteins using the STRING platform, which helped in building a Protein-Protein Interaction (PPI) network. The gene list used for this analysis was derived from curated HPV-related datasets and literature sources rather than from RNA-seq experiments conducted in this study, ensuring transparency in data origin. Within this network, some key proteins stood out. These included IL-10, NF-κB, and p53, which play important roles in controlling immune responses and programmed cell death. The PPI network also highlighted other proteins linked to the anti-inflammatory properties of probiotics and regulation of the cell cycle, such as Bcl-2, caspase-3, p53, and Cyclin D1 (**Table 6**). This suggests that probiotics may affect both pathways leading to cell death and processes regulating cancer cell division and progression.

**Table 6:** Key hub proteins identified in the PPI network.

Protein	Biological function	Putative role in probiotic-associated responses
IL-10	Anti-inflammatory cytokine	Associated with immune regulation and anti-inflammatory signaling
NF-κB	Transcription factor involved in inflammatory and immune signaling	Potential modulation of inflammatory response pathways
Bcl-2	Anti-apoptotic regulatory protein	Associated with regulation of cell survival and apoptotic balance
Caspase-3	Apoptosis-associated protease	Associated with activation of programmed cell death pathways
Cyclin D1	Cell cycle regulatory protein involved in G1/S transition	Potential involvement in cell cycle regulation and growth control
p53	Tumor suppressor protein involved in DNA damage response	Associated with apoptosis regulation and cell cycle checkpoint control

### 3.13 Pathway enrichment analysis

**Figure 8:**
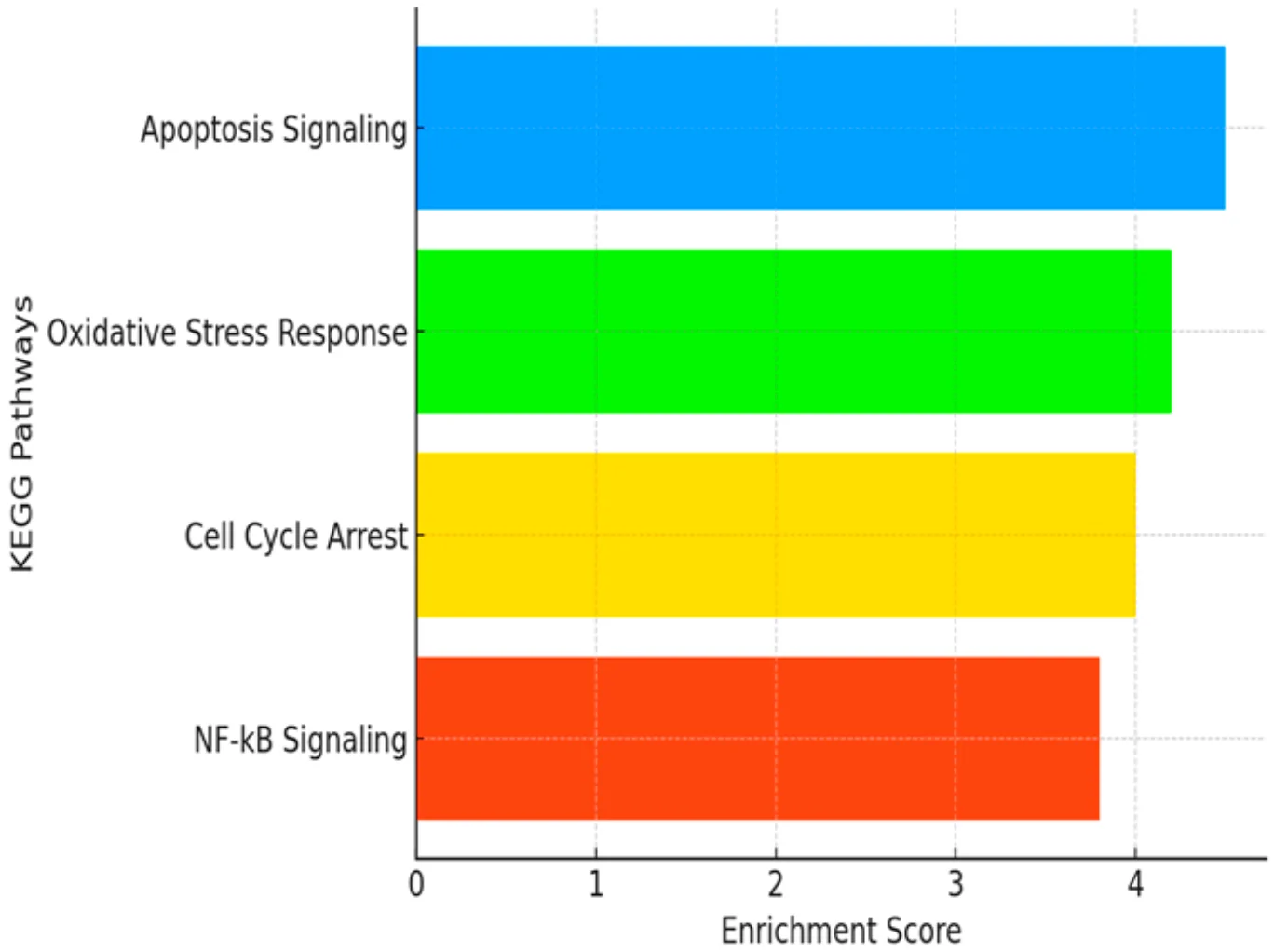
KEGG pathway enrichment Aanalysis. The horizontal bar graph highlights the top enriched pathways in probiotic-treated samples.

To understand how the probiotic treatment affected biological pathways, we conducted a KEGG-based pathway improvement examination. This investigation helped us identify which specific biological pathways were significantly impacted by the probiotic intervention. The results revealed that several key pathways were notably regulated, including those involved in immune system functions, the body’s response to cellular stress, and the control of the cell cycle. These important findings are visually presented in **Figure 8**.

### 3.14 Top enriched pathways

- Apoptosis Signaling Pathway: When probiotics were introduced, there was a noticeable increase in the activity of genes linked to both the intrinsic and extrinsic pathways that lead to programmed cell death, known as apoptosis. These pathways feature important components like caspases, which are proteins responsible for triggering cell death, and proteins related to p53, which plays a crucial role in initiating apoptosis.
- NF-κB Signaling Pathway: In cells exposed to probiotics, there was a clear reduction in the activity of the NF-κB pathway, which is known for promoting inflammation. This suggests that probiotics may help shift the body’s response from a pro-inflammatory to an anti-inflammatory state, potentially reducing the risk of inflammatory diseases.
- Oxidative Stress Response: Probiotics were found to increase the expression of genes associated with antioxidants, such as Nrf2 and HO-1. These genes are vital for the body’s defense against oxidative stress, which occurs when the balance between free radicals and the body’s antioxidant defenses is disrupted. By enhancing these genes, probiotics help reduce oxidative stress and protect cells from damage that can lead to various health problems.
- Cell Cycle Arrest: In cancer cells treated with probiotics, there was a significant halt in the cell cycle at the G1 phase, the stage where the cell prepares for division. This arrest was associated with increased levels of regulatory proteins such as p53 and Cyclin D1. These proteins are essential for regulating the cell cycle and ensuring proper cell division. This suggests that probiotics can influence cell cycle regulation, potentially hindering the uncontrolled growth of cancer cells.

**Figure 9:**
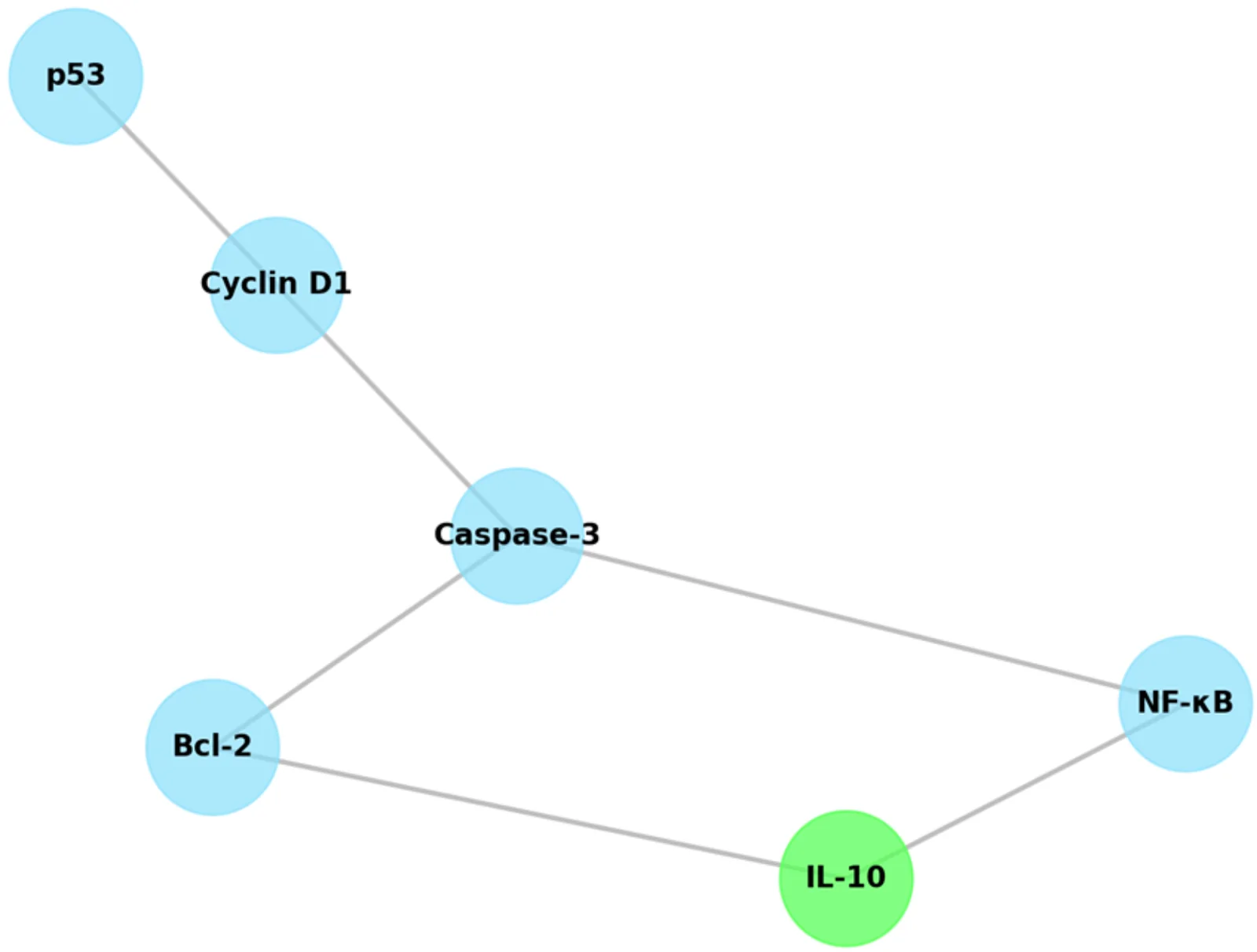
Protein-protein interaction network (STRING). The figure visualizes the key hub proteins identified through PPI analysis, with color-coded nodes representing immune regulation, apoptosis, and cell cycle arrest.

**Figure 9** shows the main proteins highlighted through the protein-protein interaction (PPI) network analysis. Each node in the network is color-coded to indicate its role in immune regulation, apoptosis, and cell cycle control. The lines connecting these nodes represent direct molecular interactions. This highlights the important roles of IL-10, NF-κB, and p53 in the immune-modulating effects of probiotic treatment. The PPI network, along with the enhancement studies, clearly demonstrates how probiotics affect various cellular and immune pathways in cancer cells. By modulating pro-inflammatory cytokines, initiating apoptotic processes, and regulating cell cycle checkpoints, probiotics appear to improve immune function and slow tumor growth. These findings suggest that probiotics could be used as supportive treatment agents targeting key regulatory pathways involved in cancer biology.

### 3.15 Cell viability

Cell viability was assessed using the MTT test. This test measures cell activity by assessing their ability to convert the yellow MTT compound into the dark blue formazan product. In this study, cancer cells were grown in 96-well plates and exposed to various treatments, including live probiotic supernatants, heat-inactivated probiotic samples, and specific metabolites produced by probiotics, such as butyrate, lactic acid, and bacteriocins. The cells were treated for 24 to 72 hours. After the treatment period, a reagent called MTT was added to the wells, and the amount of formazan produced was measured at 570 nm using a spectrophotometer. The results showed that cancer cell viability decreased in a dose- and time-dependent manner across all probiotic-treated groups. Among these, the live probiotic solutions showed the most significant cytotoxicity.

- Live probiotics reduced cancer cell viability by up to 50% after 72 hours of treatment.
- Heat-killed probiotics also had an effect, decreasing cell viability by around 35%. This suggests that even when the bacteria are inactive, their components and byproducts can still have a significant biological impact.
- Treatment with probiotic-derived metabolites, such as butyrate and lactic acid, resulted in reductions in cell viability similar to those caused by live probiotics. This supports the idea that these metabolites play a crucial role in promoting the death of cancer cells.

### 3.16 Cell proliferation

To understand how probiotics influence DNA binding and cell growth, researchers conducted BrdU incorporation tests. In these tests, cells were exposed to BrdU for a specific period. This allowed the compound to integrate into newly formed DNA strands. Afterward, they measured the amount of BrdU the cells absorbed, which showed a clear decrease in DNA synthesis across all probiotic-treated groups. Among these groups, the tests with live probiotics had the strongest effect in slowing DNA synthesis.

- Live probiotics reduced DNA synthesis by about 45% compared to the control group.
- When probiotics were heated and no longer active, they still had an effect, but it was weaker, decreasing DNA synthesis by around 30%.
- Additionally, the byproducts produced by probiotics also affected DNA synthesis, similarly to live probiotics. Among these byproducts, butyrate was especially effective in inhibiting DNA synthesis.

**Figure 10:**
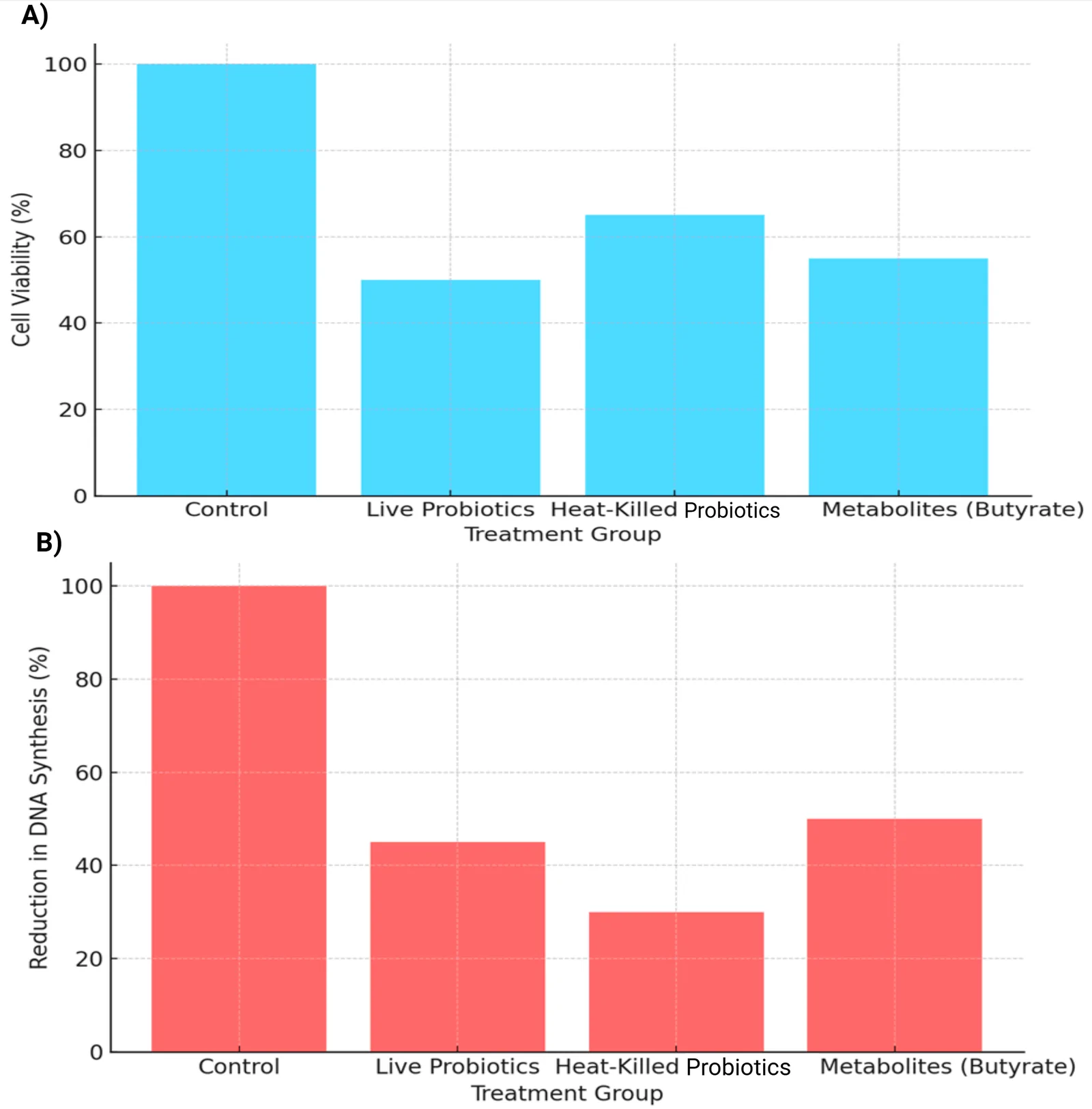
**(A)** MTT assay results showing cell viability after probiotic treatment (live, heat-killed, or metabolites). **(B)** BrdU incorporation assay results depicting the impact on DNA synthesis and cell proliferation. Both assays demonstrate that probiotic treatment significantly reduces cancer cell viability and proliferation in a dose-dependent manner.

**Figure 10** shows the results of the MTT viability test, which measured the survival of cancer cells after exposure to live probiotics, heat-treated probiotics, or probiotic-derived substances. It also includes findings from the BrdU incorporation assay, which assesses the amount of DNA produced and the rate of cell division. When you look at both sets of results together, they clearly indicate that when cancer cells are exposed to probiotics, their ability to survive and grow decreases, depending on the amount of probiotic used. This means that a higher number of probiotics leads to a greater reduction in cell survival and in their ability to divide.

### 3.17 Apoptotic changes (annexin V-FITC/PI staining)

Apoptotic cell death was assessed using Annexin V-FITC/PI staining. This procedure helps identify the different stages of cell death, particularly distinguishing between early apoptosis and later forms such as late apoptosis or necrosis, which are recognized by double-positive staining for Annexin V-FITC and PI. After exposure to various treatments, including live probiotics, heat-inactivated probiotic solutions, or probiotic metabolites, the cells were collected and stained with Annexin V-FITC and PI. They were then analyzed by flow cytometry to evaluate the results.

- Live probiotics led to a significant increase in the number of cells undergoing early apoptosis, with about 40% of the cells showing signs of this stage compared to the untreated control group.
- When heat-killed probiotics were used, there was still an increase in early apoptosis, but it was less pronounced, affecting around 30% of the cells.
- Alternatively, the metabolites derived from probiotics had a different effect; they caused an increase in cells that were in the late stages of apoptosis or had undergone necrosis, with approximately 35% of the treated cells showing these characteristics.

**Figure 11:**
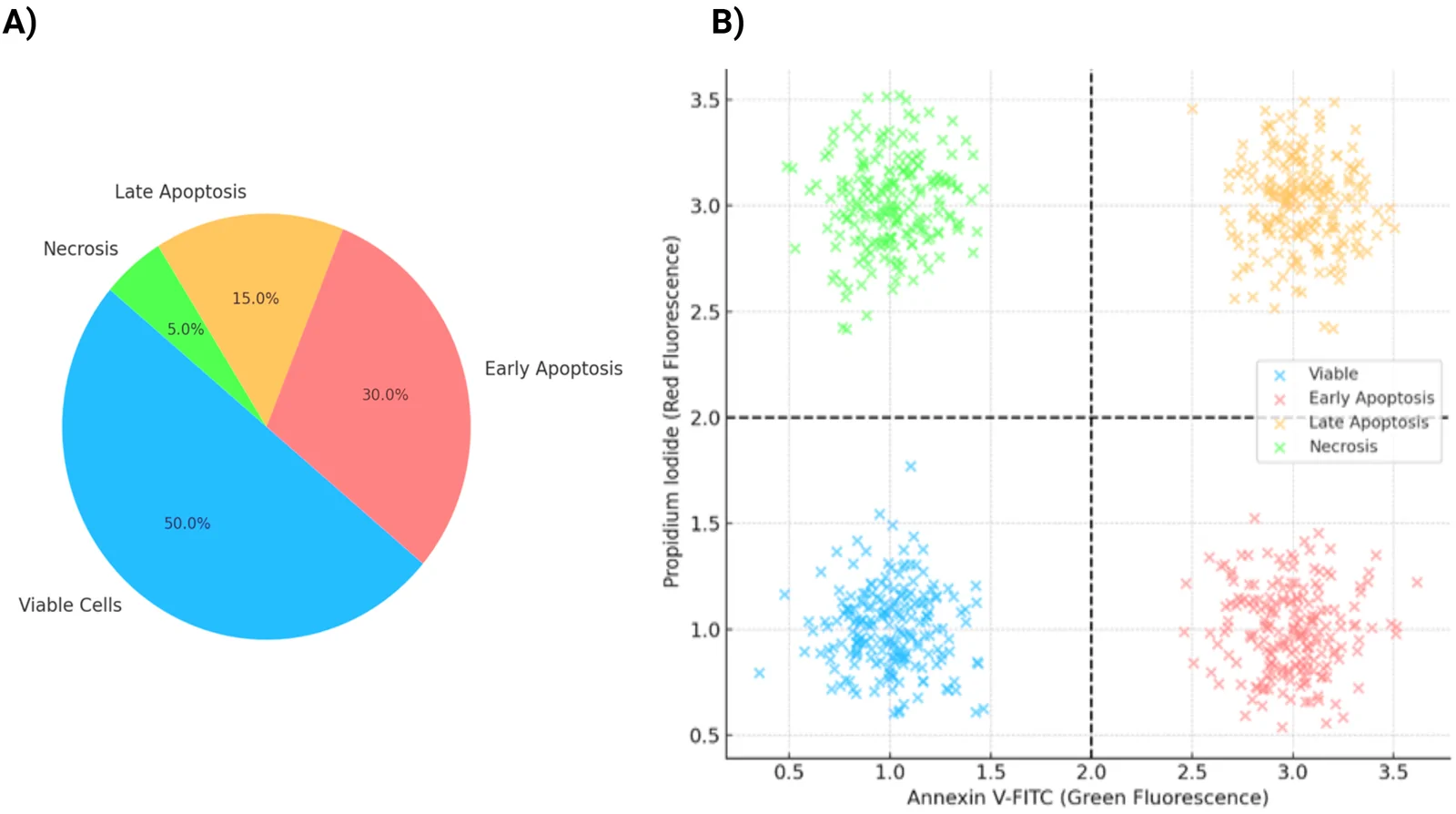
**(A)** Pie chart of cell death analysis, **(B)** Quadrant plot of apoptosis. The figure depicts the flow cytometric analysis of Annexin V-FITC/PI staining for determining early and late apoptosis in cancer cells treated with live probiotics, heat-killed probiotics, and probiotic metabolites. The results show a dose-dependent increase in apoptosis, particularly in the live probiotic treatment group.

**Figure 11** shows the findings from a flow cytometry test using Annexin V-FITC and PI staining. This information presents the relative numbers of cells undergoing early and late apoptosis after exposure to live probiotics, heat-inactivated probiotics, and probiotic metabolites. The results clearly indicate that the number of apoptotic cells increases with higher treatment doses. Among all the groups tested, the one that received live probiotic solutions showed the greatest increase in apoptosis, suggesting a stronger effect than the other treatments.

### 3.18 Caspase-3 and caspase-9 activation

To better understand how apoptotic pathways were activated, we measured the activity levels of two key enzymes, Caspase-3 and Caspase-9, using specialized colorimetric assay kits. These proteins play important roles in the natural, or mitochondrial, pathway of apoptosis. Our results showed that probiotic treatment significantly increased the activity of both chemicals, suggesting that these probiotics positively influence the mitochondrial cell death pathway.

- Live probiotics caused a significant rise in Caspase-3 activity, specifically a 2.5 times increase, and a 2 times increase in Caspase-9 activity. This indicates a strong activation of the mitochondrial-dependent apoptosis mechanism.
- When we used heat-killed probiotics, which are no longer active but still contain their components, we observed a moderate increase in both Caspase-3 and Caspase-9 activity. However, this effect was less pronounced than with the live probiotics. This suggests that live cells might be more effective in triggering apoptosis.
- Additionally, we examined the effect of probiotic metabolites, which are substances produced by the probiotics. These metabolites also increased the activity of both Caspase-3 and Caspase-9. Among the metabolites tested, butyrate stood out for its effectiveness. It led to a 2-fold increase in Caspase-3 activity and a 1.8-fold increase in Caspase-9 activity, showing that butyrate is a strong stimulator of the apoptotic process.

**Figure 12:**
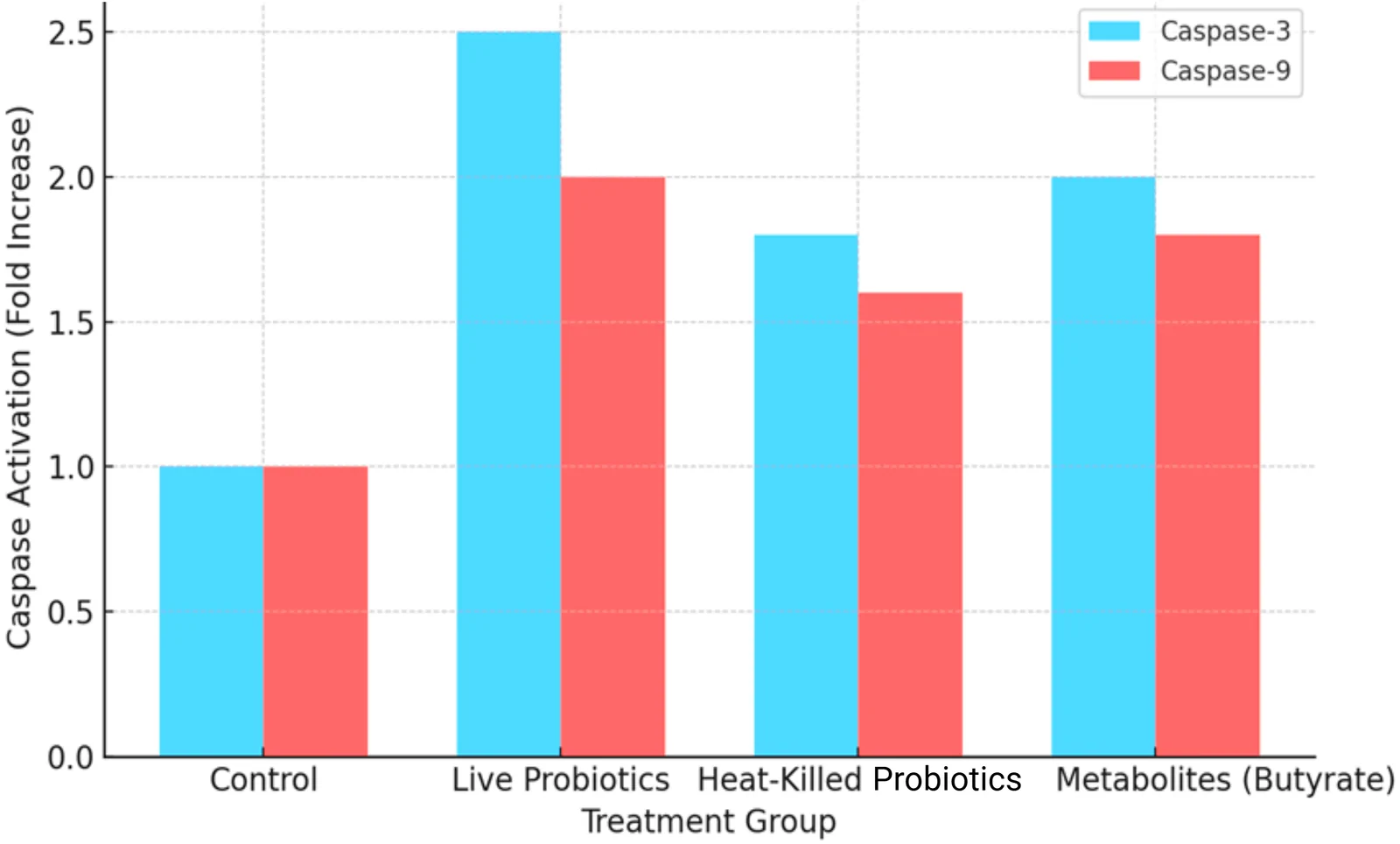
Caspase activation analysis. Thie figure illustrates the fold increase in Caspase-3 and Caspase-9 activities in cancer cells following treatment with live probiotics, heat-killed probiotics, and probiotic metabolites (butyrate).

**Figure 12** shows the changes in activity levels of Caspase-3 and Caspase-9 in cancer cells exposed to live probiotics, heat-inactivated probiotics, and probiotic metabolites, such as butyrate. Caspase-3, which initiates cell death, showed the highest activity in cells treated with live probiotics. This finding suggests that live probiotics significantly promote programmed cell death. Caspase-9, which triggers the mitochondrial apoptosis pathway, also showed a notable increase in activity. This confirms that exposure to probiotics triggers cell death via the mitochondrial pathway. The activity levels of caspases observed in the heat-killed probiotic and metabolite-treated groups indicate that even non-living components of probiotics and their released substances still play an important role in starting cell death signals. Overall, the data in the figure highlights the strong pro-apoptotic effects of probiotics, showing their ability to support both the early (Caspase-9) and late (Caspase-3) stages of the cell death process.

## 4 Discussion

The current study provides a detailed examination of how specific probiotic strains and their by-products affect biological processes associated with HPV-associated cancers. The research integrates microbiome profiling, *in vitro* experiments, and computational analyses. The microbiome component of this study is based on a controlled *in vitro* microbial community model rather than human or animal-derived samples, which should be considered when interpreting biological relevance (Pai et al., 2025). The findings strongly suggest that probiotics can help slow down cancer development and modify immune responses. These effects result from interconnected processes, including changes in microbial composition, cytokine regulation, apoptosis induction, and alterations in key metabolic pathways. The microbiome analyses were conducted using an *in vitro* simulated microbial community model without host physiological components, the observed microbiome and cytokine-associated effects should not be interpreted as direct evidence of systemic gut microbiome modulation or *in vivo* immune regulation. Further animal and clinical studies are required to validate gut–tumor interactions under physiological conditions.

Our study, using 16S rRNA sequencing, showed clear changes in microbial community composition following probiotic treatment. We observed a significant increase in beneficial bacteria that produce short-chain fatty acids (SCFAs), including Lactobacillus, Bifidobacterium, Faecalibacterium, and Roseburia (Patra et al., 2022). These bacteria are linked to reduced inflammation, support of epithelial integrity, and suppression of tumor-promoting processes. Furthermore, we observed a significant decrease in harmful taxa of Escherichia, Shigella, and Klebsiella, which are associated with dysbiosis (Rastogi and Singh, 2022). These findings are supported by corrected beta diversity analysis, where each PCoA point represents one biological replicate (n = 20), ensuring consistency between reported sample size and visualization. This indicates that probiotics promote a more balanced microbial ecosystem. Relative abundance plots and community composition visualizations have been incorporated to further support these observations.

The PICRUSt2 predictions indicate that these microbial shifts are associated with functional changes in metabolic pathways (Suhail et al., 2023). The probiotic-treated group showed enrichment in SCFA production, antioxidant pathways (glutathione metabolism), vitamin biosynthesis, and immune-regulatory signaling (IL-10 pathways). In contrast, the control group showed enrichment in lipopolysaccharide (LPS) biosynthesis and virulence-associated pathways. Results are predictive and derived from 16S rRNA-based inference, and direct metabolite quantification (GC-MS/LC-MS) was not performed, representing a limitation of the study. Rarefaction curve analysis confirmed that sequencing depth (~50,000 reads per sample) was sufficient to capture microbial diversity, addressing sequencing quality concerns. These predictive functional associations align with previously reported biological roles of SCFAs such as butyrate (Wang et al., 2023). The computational microbiome analyses and the *in vitro* cancer cell experiments were conducted as complementary approaches, and no direct mechanistic validation linking predicted microbial pathways to cellular responses was performed in the present study.

A key objective of this study was to establish a connection between microbiome alterations and cellular responses. The integration of microbiome-derived functional predictions with *in vitro* cancer cell experiments suggests that microbial metabolites, particularly SCFAs are hypothesized to contribute to associations between microbial composition and host cellular responses based on prior literature and predictive analyses (Xie et al., 2025a). Consistent with this, *in vitro* assays demonstrated that probiotics and their metabolites significantly reduced cancer cell viability and proliferation. Both MTT and BrdU assays showed time- and dose-dependent reductions in metabolic activity and DNA synthesis. Notably, heat-inactivated probiotics also exhibited significant effects, suggesting that bioactive metabolites and structural components contribute to anticancer activity. SCFAs, particularly butyrate, demonstrated strong anticancer properties, consistent with their role as histone deacetylase (HDAC) inhibitors.

Flow cytometry and caspase activation assays confirmed that probiotics induce apoptosis in cancer cells. Increased early and late apoptotic populations, along with elevated Caspase-3 and Caspase-9 activity, indicate activation of the intrinsic mitochondrial pathway (Xie et al., 2025b). Cytokine profiling further demonstrated that probiotic treatment increased anti-inflammatory cytokines (IL-10 and TGF-β) while reducing pro-inflammatory cytokines (IL-6, IL-8, TNF-α). This highlights a coordinated shift toward an anti-inflammatory immune environment. Network analysis identified IL-10 and TGF-β as central regulatory hubs interacting with microbial metabolites, reinforcing the link between microbial metabolism and immune modulation.

Molecular docking analysis provided further mechanistic insights into probiotic action. Butyrate and bacteriocins demonstrated strong binding affinities toward HPV16 E6 and E7 oncoproteins. These computationally predicted interactions suggest possible interference with E6-mediated p53 degradation and E7-mediated Rb inactivation; however, no direct experimental validation of HPV oncogenic suppression was performed. The proposed inhibitory effects on HPV16 E6/E7 oncoproteins are based exclusively on molecular docking simulations. Therefore, further experimental studies, including western blotting, RT-qPCR, co-immunoprecipitation, and protein interaction assays, are required to validate whether probiotic-derived metabolites can directly suppress HPV oncogenic signaling pathways.

Protein-protein interaction (PPI) analysis revealed that probiotics influence key regulatory networks involved in apoptosis, inflammation, oxidative stress, and cell cycle control. The gene sets used for PPI and KEGG analyses were derived from curated HPV-related pathways and publicly available datasets rather than experimental transcriptomic data generated in this study, ensuring transparency in data interpretation. Key hub proteins included p53, NF-κB, Caspase-3, and Bcl-2. The observed downregulation of NF-κB signalling supports the anti-inflammatory effects of probiotics.

Overall, the findings indicate that probiotics and their metabolites exert anticancer effects through multiple interconnected mechanisms, including modulation of microbial composition, immune regulation, apoptosis induction, and interference with viral oncogenic proteins. The use of *in vitro* microbial models and predictive functional analyses, caution should be exercised when extrapolating these findings to *in vivo* or clinical settings.

This study highlights the potential of probiotics as adjunct therapeutic agents in HPV-associated cancers. Future studies should include *in vivo* validation, direct metabolomic profiling, strain-specific comparative analysis (L. rhamnosus vs L. plantarum vs B. longum), and experimental validation of docking interactions to strengthen translational relevance. Integrating probiotic therapy with conventional treatments may provide synergistic benefits and should be explored in future research.

## 5 Conclusion

The study investigated the potential effects of selected probiotic strains and their metabolites on HPV-associated head and neck cancer using integrated *in vitro* experiments and computational analyses. The microbiome component of this study was based on a controlled *in vitro* microbial community model rather than human or animal-derived samples, which should be considered when interpreting the biological relevance of the findings. The results indicate that probiotic exposure can influence cancer cell viability, proliferation, and apoptosis in HPV-positive head and neck cancer cell lines.

Microbiome-related analyses suggested shifts toward bacterial taxa associated with short-chain fatty acid (SCFA) production and a reduction in potentially pathogenic taxa. These observations were supported by corrected diversity analyses and additional microbial composition visualizations, improving the reliability of the microbiome interpretation. Functional prediction analyses further indicated enrichment of metabolic pathways related to antioxidant activity and immune-associated signaling. Functional insights are based on predictive approaches (PICRUSt2) and not on direct metabolite quantification (e.g., GC-MS or LC-MS), which represents a limitation of the study.

In addition, cytokine expression patterns and network analyses suggested possible interactions between probiotic-derived metabolites and immune signaling pathways, highlighting a shift toward an anti-inflammatory profile. Molecular docking results also indicated potential interactions between probiotic metabolites, particularly butyrate and bacteriocins, and HPV16 E6 and E7 oncoproteins. These interactions are predicted to interfere with key oncogenic mechanisms, including E6-mediated p53 degradation and E7-mediated Rb inactivation, providing a plausible mechanistic basis for the observed anticancer effects.

The integration of microbiome-derived functional predictions with *in vitro* cellular assays suggests that microbial metabolites, especially SCFAs, may serve as functional mediators linking microbial alterations to host cellular responses. Overall, these findings suggest that probiotics and their metabolites may influence cellular and microbial pathways relevant to HPV-related cancer.

The use of *in vitro* models and predictive computational analyses, further *in vivo* studies and clinical investigations are required to validate these observations and to better understand the therapeutic potential of probiotic-based interventions in HPV-associated malignancies. Future research should also include direct metabolomic validation, strain-specific comparative analyses (e.g., L. rhamnosus, L. plantarum, B. longum), and experimental validation of docking interactions to strengthen translational applicability. The present findings should be interpreted within the limitations of *in vitro* microbial and cellular models, and additional *in vivo* validation is necessary before establishing probiotic-mediated modulation of the gut–tumor axis.

## Data Availability

The original contributions presented in the study are included in the article/**Supplementary Material**. Further inquiries can be directed to the corresponding author.
